# Leveraging Patient-Derived Organoids for Personalized Liver Cancer Treatment

**DOI:** 10.7150/ijbs.96317

**Published:** 2024-10-07

**Authors:** Jianhua Rao, Chao Song, Yangyang Hao, Zaozao Chen, Sidu Feng, Shihui Xu, Xiaoyue Wu, Zhengfeng Xuan, Ye Fan, Wenzhu Li, Junda Li, Yong Ren, Jian Li, Feng Cheng, Zhongze Gu

**Affiliations:** 1 Hepatobiliary Center of The First Affiliated Hospital, Nanjing Medical University; Research Unit of Liver Transplantation and Transplant Immunology, Chinese Academy of Medical Sciences, Nanjing, China.; 2 State Key Laboratory of Digital Medical Engineering, Southeast University, Nanjing, China.; 3 Key Laboratory of DGHD, MOE, School of Life Science and Technology, Southeast University, Nanjing, China.; 4 School of Biological Science & Medical Engineering, Southeast University, Nanjing, China.; 5 State Key Laboratory of Translational Medicine and Innovative Drug Development, Jiangsu Simcere Diagnostics Co, Nanjing, China.; 6 Jiangsu Avatarget Co, Suzhou, China.; 7 Jiangsu Institute for Health and Sport (JIHS), Nanjing, China.; 8 Institute of Medical Devices (Suzhou), Southeast University, Nanjing, China.

**Keywords:** primary liver cancer, organoid, personalized treatment, drug screening

## Abstract

Primary liver cancer (PLC) is a primary cause of cancer-related death worldwide, and novel treatments are needed due to the limited options available for treatment and tumor heterogeneity. 66 surgically removed PLC samples were cultured using the self-developed 2:2 method, and the final success rate for organoid culture was 40.9%. Organoid performance has been evaluated using comprehensive molecular measurements, such as whole-exome and RNA sequencing, as well as anticancer drug testing. Multiple organoids and their corresponding tumor tissues contained several of the same mutations, with all pairs sharing conventional TP53 mutations. Regarding copy number variations and gene expression, significant correlations were observed between the organoids and their corresponding parental tumor tissues. Comparisons at the molecular level provided us with an assessment of organoid-to-tumor concordance, which, in combination with drug sensitivity testing provided direct guidance for treatment selection. Finally, we were able to determine an appropriate pharmacological regimen for a patient with ICC, demonstrating the clinical practicality in tailoring patient-specific drug regimens. Our study provides an organoid culture technology that can cultivate models that retain most of the molecular characteristics of tumors and can be used for drug sensitivity testing, demonstrating the broad potential application of organoid technology in precision medicine for liver cancer treatment.

## Introduction

Liver cancer, a leading global healthcare challenge, is one of the most common malignancies, and its incidence is increasing annually 1. There are more than 1 million cases of liver cancer predicted to occur each year by 2025 2. Primary liver cancer (PLC) represents a heterogeneous group of tumors with apparent histological characteristics and clinical outcomes, among which hepatocellular carcinoma (HCC) is the most common subtype, followed by intrahepatic cholangiocarcinoma (ICC) 3. Studies have demonstrated that PLC has various intricate and diverse alterations involving somatic mutations, DNA copy number variations (CNVs), a high degree of aneuploidy, and epigenetic variations, which all lead to varying degrees of HCC 4. This molecular heterogeneity in PLC highlights the need for personalized treatment.

Although various pioneering advances have been achieved in cancer biology since 2D culture was introduced 5, the key characteristics of growing tumors have failed to be recapitulated, especially for three-dimensional (3D) organizations 6, 7. In recent years, organoids have emerged as 3D miniature structures that are near-physiological culture systems of primary, nontransformed tissues that can accurately recapitulate the histological architecture and functions of the parent tissue 8. In order to study the development of human diseases, organoids from various tissues, including the kidney, brain, stomach, and retina, have been produced from pluripotent stem cells 9. Organoids are considered potential disease models for various studies, because of their biology and their efficacy in evaluating drug responses in vitro 10. However, to date, studies on human liver cancer organoids are limited.

In a study on the development of long-term patient-derived organoid (PDO) culture from liver tumors, Broutier et al. reported that the organoid culture retained the histological architecture and genetic traits of the original tumor and distinguished among disparate tumor tissues and subtypes 11. In a similar study, HCC organoids from tumor needle biopsies were found to maintain the genetic heterogeneity, gene expression patterns of tumor markers, and morphologies of the corresponding original tumors 12. Accordingly, the development of PLC-derived organoids (PLCOs) is important and can help elucidate the molecular mechanisms of HCC to explore individualized treatments. Herein, we developed and evaluated PLCOs, demonstrating the accurate recapitulation of the biological features and genetic traits of parental tumor tissues. We further utilized PLCOs to predict patient-specific drug sensitivity patterns and provided personalized treatment guidance.

## Materials and methods

### Sample collection

The samples were obtained from 66 patients with PLC who were surgically treated at Jiangsu Province Hospital in Nanjing, China. An independent histopathologist confirmed the diagnosis of each patient based on routine hematoxylin and eosin (H&E)-stained sections. The samples were processed according to the Health Technology Assessment guidelines, and each sample was divided into four parts for organoid culture, histological analysis, and DNA and RNA isolation. Written consent was provided by all the patients. The present study was approved by the Institutional Review Board of Jiangsu Province Hospital and tissue acquisition was performed after receiving ethical approval.

### Tissue digestion, organoid quantification and derivation

The liver tissues were divided into four pieces, with two pieces snap frozen and stored at -80°C for DNA and RNA sequencing, one piece fixed in 4% polyformaldehyde for histopathological analysis and immunohistochemistry, and the remainder processed for organoid culture. The liver tissue was washed several times using phosphate-buffered saline (PBS; containing 1% penicillin/streptomycin) after removing the blood vessels and fat. Next, the tissue was minced and incubated with the digestion solution for 2 h at 37°C. The digestion solution was renewed, and the sample was left for 2-4 h according to the degree of liver fibrosis. This two-step digestion facilitated fibrinolysis, and the digestion was stopped when no tissue pieces could be found on visual inspection.

The suspension was then filtered using a 100-µm nylon cell filter, and the filtrate was centrifuged at 1500 rpm for 5 min. The precipitate was resuspended in PBS, transferred to a 2-mL Eppendorf tube and again centrifuged at 1500 rpm for 5 min. Finally, the cell precipitate was evenly mixed with the Matrigel and seeded in a 24 well plate. Following the solidification of the Matrigel, 500 µL of liver tumor organoid medium (Jiangsu Avatarget Biotechnology) was added to individual wells, and the plates were transferred to humidified 37°C/5% CO2 incubators.

To better understand the biological characteristics and experimental results of organoids, the analysis of their digestion and subsequent quantification is crucial. Organoids can be visually observed and counted under brightfield microscopy, but manual screening is time-consuming, labor-intensive, and prone to subjective errors. Deep learning-based approaches can rapidly quantify and compute the number and diameter parameters of digested organoids, reducing human intervention and providing more accurate measurements. In this study, we combine Yolov8 with the SAHI algorithm 13, 14. Yolov8 is known for its high precision in object detection tasks, and its highly optimized network architecture enables fast inference. However, detecting extremely small and densely packed organoids poses a major challenge. Extremely small organoids are represented by only a few pixels in images, while densely packed ones suffer from severe occlusion and lack sufficient detail, making them difficult to detect with conventional detectors. The integration of the Slice-Assisted Hypothesis Inference (SAHI) algorithm tentatively attempts to addresses this issue by providing a general slicing-assisted inference and fine-tuning pipeline for dense object detection. By leveraging slice assistance, it can push the limits of object detection methods to some extent, which may help improve the accuracy of counting and detecting small targets like digested organoids. This holds significant potential for daily organoid cultivation, drug sensitivity testing, and other processes (Figure S1). Nevertheless, further investigation and validation are needed to assess the effectiveness of this method in rapidly quantifying and computing the number and diameter parameters of digestive organoids, topics which we plan to report on in future studies.

The medium was changed every 3-4 days, and the organoids were passaged every week. The organoids were collected from the basement membrane extract (BME) using PBS and digested with TrypLE (Thermo Fisher), for 2 min. The suspension was centrifuged, and the precipitate was plated and transferred following the abovementioned methodology.

To preserve the tumor organoids, the organoids were collected from the BME using PBS and centrifuged at 1500 rpm for 5 min. The precipitate containing the organoids was mixed with protein-free, CD cell cryopreservation medium (ExCell Bio) and frozen according to standard procedures. The specific composition of the culture media is summarized in Table S1.

### Histology and staining

The organoids and tumor tissues were both fixed with 10% neutral-buffered formalin at room temperature (25℃) for 0.5 h and 24 h, respectively, and then embedded in paraffin to obtain formalin-fixed paraffin-embedded (FFPE) samples. First, the tissues were processed using a graded ethanol series followed by xylene treatment. Next, the tissues were embedded in paraffin and cut into sections. H&E and immunohistological staining of tissues were subsequently performed. Forthe immunofluorescence experiment, PBS was used to rehydrate the organoids after formalin fixation. Regarding immunohistological staining, citrate sodium solution (pH = 6) was used to deparaffinize the FFPE organoids, and the deparaffinized sections were subjected to antigen retrieval. The sections were supplemented with 3% bovine serum albumin and 0.5% Triton and incubated in Tris-buffered saline solution for 1 h to decrease background nonspecific staining. Finally, the sections were incubated overnight with appropriately diluted primary antibodies at 4°C.The organoids were washed with PBS after overnight incubation for 2-3 days at 4°C and then incubated withfluorophore-conjugated secondary antibodies.

### Organoid evaluation assay

After 2-3 weeks of isolation, a Leica M80 stereoscope was used to take pictures of full-drop BMEs obtained from each sample culture to evaluate the organoid formation efficiency, and all the viable organoid structures were marked.

For the drug sensitivity assays, enzymatic disassociation with TrypLE (Thermo Fisher) was first applied to dissociate the organoids into two-five cell clumps. Next, 250 µL of expansion medium, containing 500 clumps, from each sample was individually plated in a 48-well cell culture plate to perform the cell viability assay. The medium was changed three times a week for 3 weeks. The number of viable cells was evaluated based on the de novo capability of generating organoids. Representative images of viable cells were taken 2-3 weeks after starting the treatment.

### Drug screening of organoids

The organoids were resuspended in Matrigel, and 2-µL suspensions were individually dispensed in 384-well plates. Thereafter, the organoids were cultured for 2 d, after which the original culture media was replaced with media containing various concentrations of drugs (0.01 µM, 0.1 µM, 0.3163 µM, 1 µM, 3.163 µM, or 10 µM). At different time points (days 1, 4, and 7), the growth status of the organoids treated with different drugs was continuously observed and recorded using a high-content imaging analysis system (Avatarget). Finally, according to the manufacturer's instructions, cell viability was assessed in each well with CellTiter-Glo (catalog G9683, Promega) following 7 days of culture. Drugs that induced <50% viability were selected for further study 15.

### Whole-exome sequencing (WES)

The genomic DNA from the tumor tissues and organoids was extracted using the Qiagen DNAeasy kits (Qiagen, USA). The Qubit 2.0 Fluorometer (Life Technologies, USA) was used for quantifying DNA. The KAPA Hyper Prep Kit (KAPA Biosystems, USA) was used to prepare the sequencing libraries by amplifying the captured DNA using KAPA HyperExome Probes (Roche, USA). Next, paired-end sequencing was performed on an Illumina NovaSeq 6000 (Illumina Inc., USA). Burrows-Wheeler Aligner software was applied to align the sequencing reads to the human reference genome (hg38). After removing the PCR duplicates and correcting the base quality, the BAM files were obtained. Finally, sample pairs of control-tumor tissue or control-organoid were constructed. Somatic mutations were identified by MuTect2 (v4.3.0.0) with default parameters, and CNVs were detected using CNVkit 16.

### RNA sequencing

A RNeasy FFPE Kit (Qiagen, Hilden, Germany) was used for isolating RNA from the organoids, and the Smartseq2 method was used for preparing the RNA libraries. RNA sequencing was performed using the Illumina NovaSeqTM 6000 system. FastQC software (v0.12.1) was used to evaluate the quality of the original FASTQ files. Using parameters such as --length 36, a quality threshold of --quality 25, and a trimming stringency of --stringency 3, trim_galore software (v0.6.10) was used to trim and filter the initial FASTQ files, efficiently eliminating low-quality nucleotides and adapter sequences. STAR (v2.7.10a) was modified to align the trimmed FASTQ files to the Ensembl version 107 reference genome (hg38). The RSEM tool (v1.3.1) was used to compute gene expression abundance based on matched BAM files to generate an expression profile.

### Transcriptome data analysis

Analysis of differential gene expression between organoids and tumors was performed using the Limma package. The false discovery rate (FDR) was controlled with the Benjamini algorithm, and the threshold p--value was determined. Genes with an FDR<0.05 were considered differentially expressed genes (DEGs). Subsequently, functional enrichment analyses of DEGs between the organoids and tumor tissues were performed in the gene ontology (GO) terms and Kyoto Encyclopedia of Genes and Genomes (KEGG) pathways.

The single-cell transcriptomic data of GSE146115 17 were obtained from Gene Expression Omnibus (GEO) (https://www.ncbi.nlm.nih.gov/geo/). The GSE146115 dataset contains 3134 cells from 4 patients covering five cell types: tumor cells, stromal cells, and immune cells (B, CD8+ T cells, monocytes/macrophages). The cell annotation information was obtained from the TISCH database 20. Based on the single-cell transcriptomic data, the tumor cell-specific genes from each sample were extracted using the FindMarkers function from the R package Seurat (v4.3.0). Twenty HCC cell lines from the Cancer Cell Line Encyclopedia (CCLE) 18, 19 (https://depmap.org/portal/), as well as 68 HCC-derived organoids (HCCOs) from GSE182593 (24 HCCOs) 21 and the biosino NODE database (OEP003191) (44 HCCOs) 22 were downloaded for comparison. We calculated the similarity of the above cell lines, HCCOs, and our HCCOs and tumor tissues to the expression patterns of real tumor (malignant) cells by the Spearman correlation test. To avoid the influence of functional genes in other nonmalignant cells, the similarity was calculated only for tumor cell-specific genes. To facilitate the comparison of single-cell data with other bulk data, single-cell expression profiles were first converted into pseudotissue data. To remove the effects of different measurements, batches, and quantiles, intersecting genes from different datasets (including single cells, cell lines, organoids, and tumors) were selected, gene expression values within the same sample were ordered, and the original expression values were replaced in order. Additionally, differential expression analysis of the single-cell expression profile was performed to extract the characteristic genes of various immune cells using the FindMarkers function. To estimate the content of various cell types in the tissues, the overall degree of expression of these characteristic genes was assessed using the ssGSEA algorithm in the gsva function of the GSVA package (v 1.46.0).

### Statistical analyses

The statistical analyses were conducted using R (v 4.2.2) and GraphPad Prism software. Student's two-tailed t--test or the Wilcoxon signed-rank test was performed to compare differences between organoids and tumors. The normality and equal distribution of variance were assumed among different groups when Student's two-tailed t--test was performed. A p--value of <0.05 was considered to indicate statistical significance.

## Results

### Establishment and histopathological evaluation of organoids

In total, 66 surgically resected samples were obtained from untreated patients with PLC between December 2020 and December 2021. Each sample was divided into several parts for organoid culture, histological evaluation, and DNA and RNA isolation.

Due to the typical presence of fibrosis in liver cancer tissues, we improved the cultivation methodology of liver cancer organoids based on the 2:2 method, which consists of dual-step digestion of tumors and the use of two distinct culture media for primary and passaged tissues to maximize cultivation efficiency. The initial phase involved preliminary digestion to obtain a specific cell quantity, followed by secondary digestion until no tissue remnants were remained. In addition to conventional culture media, nicotinamide and Rspo-1 conditioned media were used for primary culture. After approximately 7 days, during passage culture, nicotinamide and Rspo-1 conditioned media were removed, and BMP4 was added. The progress of organoid growth was consistently monitored and documented through visual inspection.

Of the 66 samples, 27 PLCOs were successfully cultured, with an organoid generation success rate of 40.9% (27/66). Due to an insufficient number of cells in organoid cultures, 20 pairs of organoids and tumor tissues were matched for WES, of which 6 samples were selected for drug sensitivity testing. Nevertheless, 2 samples were obtained from the same patient (No. 35); thus, 19 patients were enrolled for analysis, including 13 patients with HCC, 3 patients with of ICC, and 3 other patients. The baseline characteristics of the 19 patients are summarized in Table S2.

**Figure 1:**
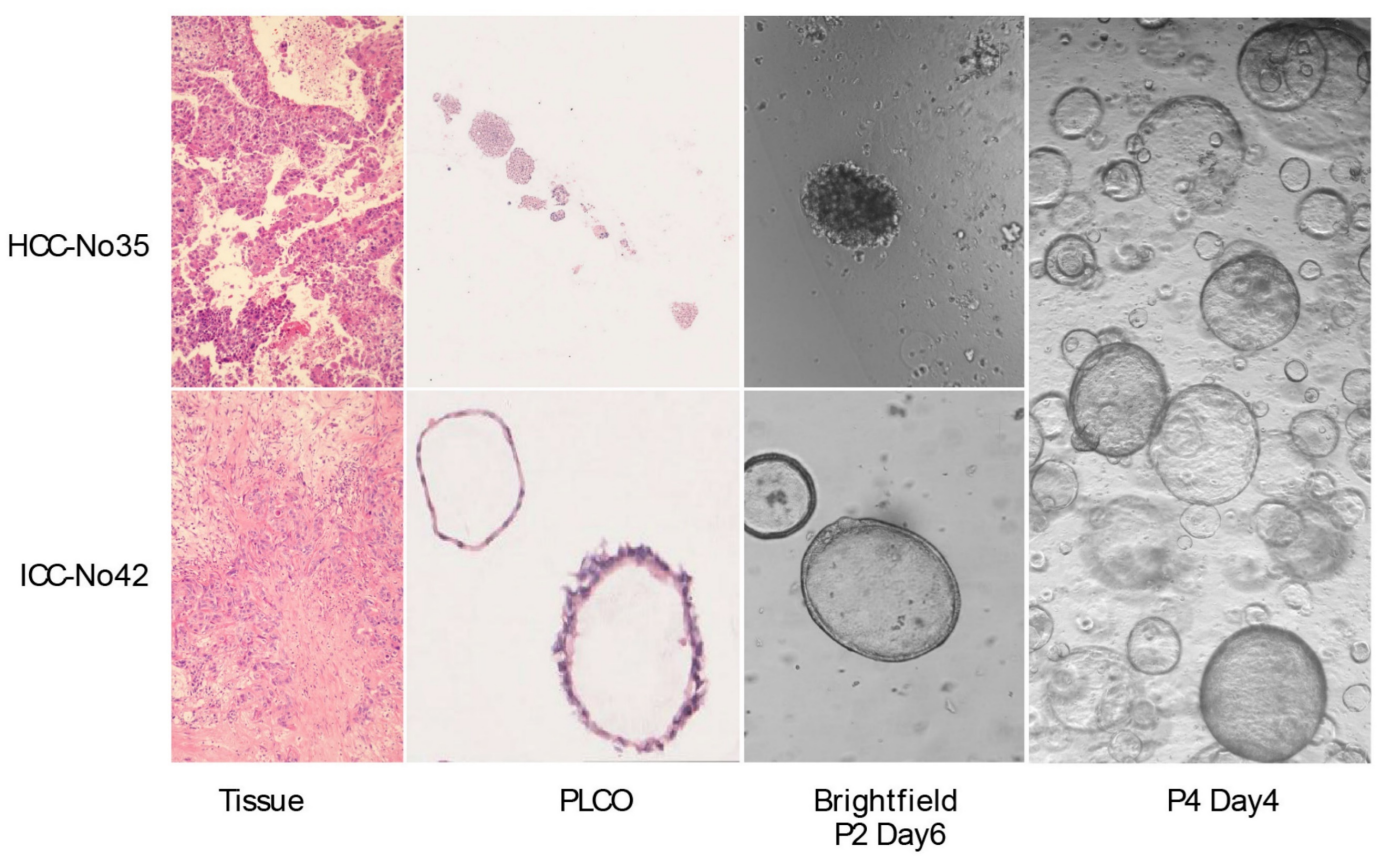
** Patient-derived primary liver cancer (PLC) organoid cultures.** Representative H&E staining of the organoids and corresponding tumor tissues. H&E staining of HCC No. 35, and ICC No. 42 tissues and PLCOs (top and second row, scale bar: 100 µm) and brightfield microscopy images of second-generation (P2) organoids on day 3. No. 42 was well cultured to the fourth generation (P4), showing good growth and retaining the organoid structure.

To observe whether the PLCOs preserved the histopathological characteristics of the parental tumor tissue, we compared the H&E staining of the organoids with that of the tumor tissues. After long-term growth, some organoids can last up to 3 months. The culture medium was changed every 7-10 days and organoid growth was continuously detected using a brightfield microscope. The results showed that the organoids exhibited histological characteristics and formed compact structures similar to those of tumor tissues. HCC exhibited typical hepatic cell cords and pseudoglandular rosettes, and ICC exhibited luminal structures (Figure 1, Figure S2).

### Recapitulation of genomic changes by PLCOs

**Figure 2:**
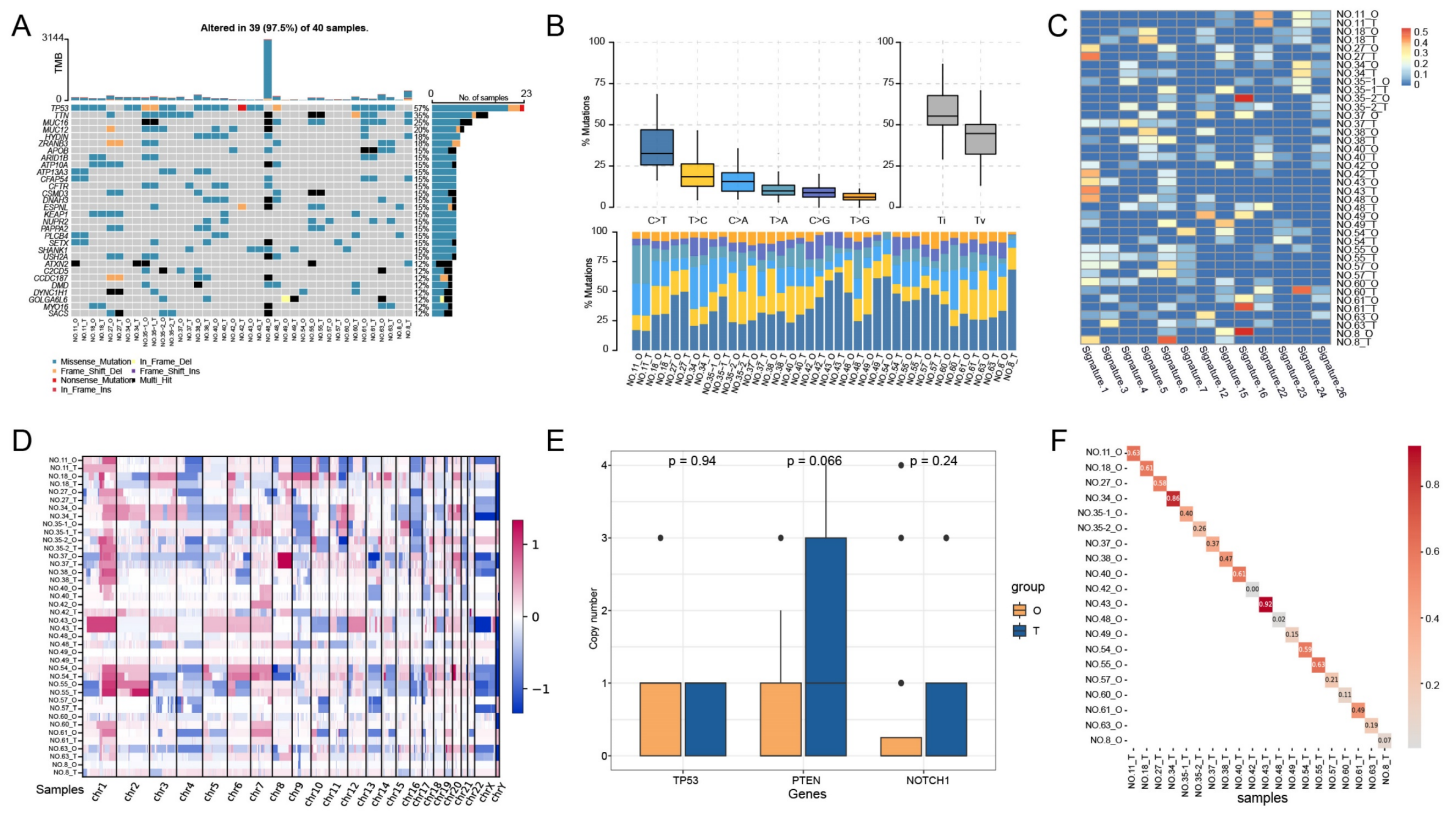
** Genomic alterations of PLCOs vs. tumor tissues based on whole-exome sequencing (WES).** (A-D) Somatic mutational landscape (A), transition and transversion (B), distribution of major signatures (C), and a visualized heatmap of copy number variations (CNVs) (D) of all samples using WES. (E) The copy numbers of several genes associated with the development of liver cancer were compared between PLCOs and tumors, with p values for differences calculated using the Wilcoxon test. (F) Correlation analysis of PLCOs vs. tumor tissues at CNV level.

To investigate whether the PLCOs maintained the mutational landscape of the parental tumor tissue, we performed a WES analysis of PLCOs and compared the results with those of the corresponding tumor tissues. Among the 20 pairs of PLCOs and tumor tissues sequenced, *TP53* was identified as the most common mutated gene, with a mutational frequency of 57%, followed by *TTN* (35%) and *MUC16* (20%) (Figure 2A). Nevertheless, the mutational type of *TP53* was the same in multiple matched PLCOs and tumor tissues, suggesting a high consistency of *TP53* between PLCOs and tumor tissues. The mutation profiles of these high-frequency mutated genes in sample 43 were highly consistent between PLCOs and tumor tissues. By analyzing the transition and transversion of all the samples, we found that the overall distribution of the 6 different transitions was similar between the PLCOs and tumor tissues of No. 11, No. 18, No. 27, No. 34, No43, and No. 55 (Figure 2B), suggesting that the PLCOs were consistent with the parental tumor tissue in terms of mutations to some extent. The distribution of 30 mutational signatures in the Catalog of Somatic Mutations in Cancer (COSMIC) (https://cancer.sanger.ac.uk/signatures/signatures_v2) was examined in organoids and tumors (Figure 2C). The mutational signatures of paired organoids and tumor tissues from No. 27, No. 42, and No. 43 were consistent with each other because they shared the mutational signature 1. Signature 1 is the result of an endogenous mutational process triggered by spontaneous deamination of 5-methylcytosine and is associated with a small number of small insertion and deletion mutations in most tissue types. Additionally, the heatmap of CNVs also exhibited a certain consistency of the organoids with the parental tumor tissue (Figure 2D). We compared the copy numbers of several genes that have been linked to the development of liver cancer in organoids and tumor tissues. We compared the copy numbers of several well-known genes associated with the occurrence and progression of liver cancer and found that *TP53* exhibited similar copy number levels in PLCOs and tumor tissues, while the copy number levels of *PTEN* and *NOTCH1* in PLCOs were slightly lower than those in tumor tissue (Figure 2E). *PTEN* is frequently lost in hepatocellular carcinoma and is associated with immune evasion and poor outcomes 23. Our PLCOs did also exhibited a lower copy number, whereas the tumor tissue exhibited a greater copy number than did the PLCOs, likely because other cells in the tumor microenvironment influence the average copy number. *NOTCH1*, a gene associated with tumor metastasis, has been reported to be differentially expressed between tumor border cells and tumor core cells 24. Differences in copy number between tumor tissues and organ tissues may be only minor differences caused by intratumor heterogeneity in the metastatic ability of PLCOs versus tumor tissues. Copy number correlation analysis revealed strong correlations between many PLCOs and paired tumor tissues, with 12 of 20 pairs maintaining a copy number concordance greater than 30%, particularly No. 43 (r = 0.92) and No. 34 (r = 0.86) (Figure 2F).

### Recapitulation of expression profiles by PLCOs

**Figure 3:**
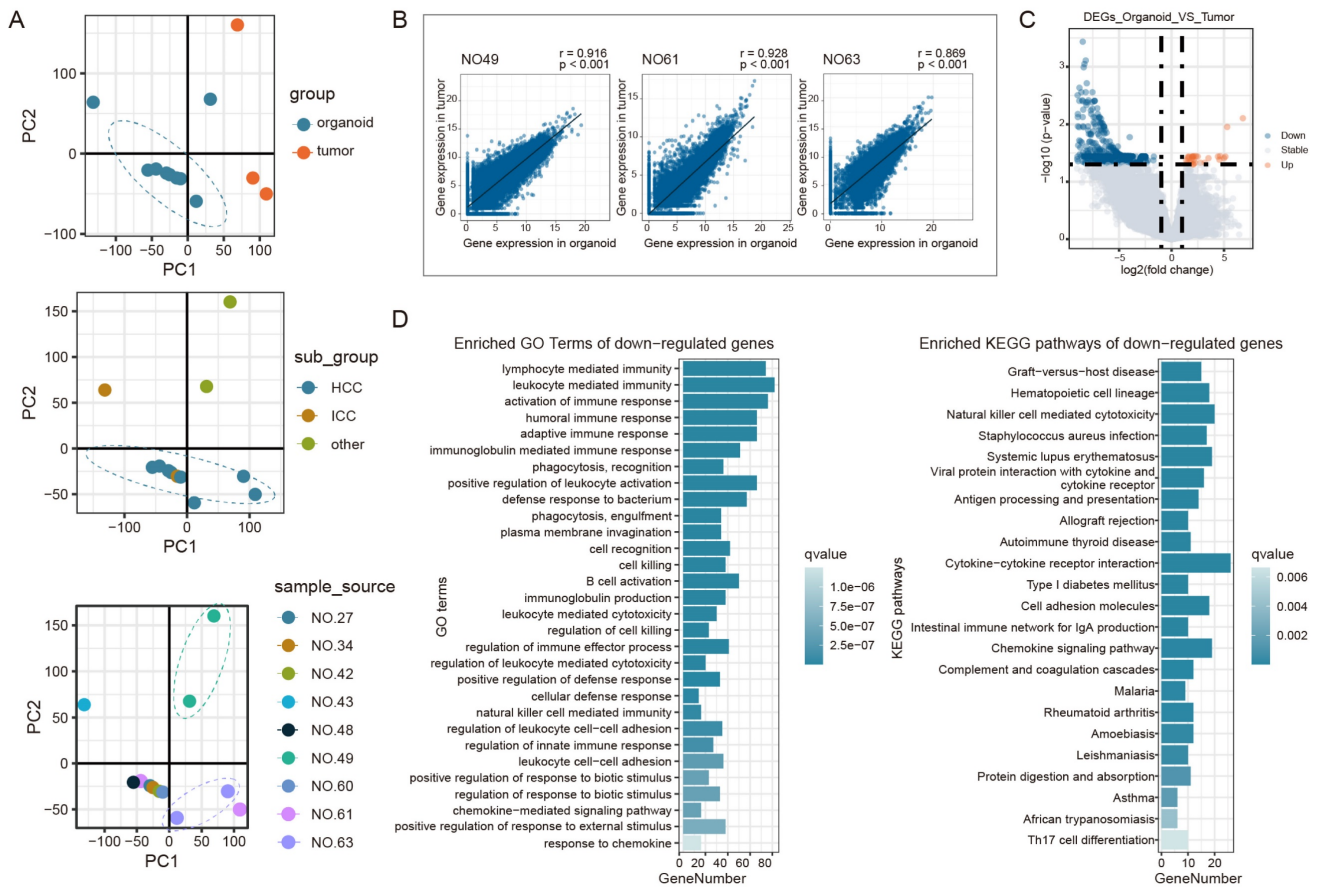
** Expression profiles based on RNA sequencing.** (A) PCA analysis of PLCOs and tumor tissues. (B) Correlation analysis of the expression levels between PLCOs and tumor tissues based on RNA sequencing. (C) Volcano plot of DEGs between PLCOs and tumors. (D) The GO and KEGG enrichment results of downregulated DEGs.

In previous studies, researchers characterized the gene expression patterns of PLC subtypes, including HCC, ICC, and combined hepatocellular cholangiocarcinoma 25, 26. Accordingly, to further examine the organoid cultures, RNA sequencing was performed to compare the expression profiles of PLCOs with those of their corresponding tumor tissues. Transcriptome sequencing was performed on three pairs of tumors and paired PLCOs from NO.49, NO.61, and NO.63, and PLCOs from samples NO.34, NO.43, NO.27, NO.48, NO.60, and NO.42. Principal component analysis (PCA) revealed global transcriptome differences between PLCOs and tumor tissues and between different cancer subtypes while maintaining some individual specificity; that is, tumor tissues and PLCOs from the same patient were more similar (Figure 3A). Correlation analysis further demonstrated that gene expression in PLCOs was strongly correlated with that in their corresponding parental tumor tissues (Spearman's correlation coefficients (r) = 0.916, 0.928, and 0.869, respectively) (Figure 3B). This suggests that paired tumor tissues and organoid transcriptional profiles are highly similar. We also conducted RNA-seq on the organoids without medium optimization. The results clearly showed that the gene expression patterns of the organoids cultured using optimised different optimized media methods were closer to the original tissues (Figure S3 and Figure S4). By differential expression analysis of HCCs from organoid and tumor tissues, 1018 DEGs were identified, including 28 upregulated genes and 990 downregulated genes (Figure 3C). Enrichment functional analysis indicated that the downregulated genes were enriched mainly in immune-related pathways while the upregulated genes were not enriched (Figure 3D).

**Figure 4:**
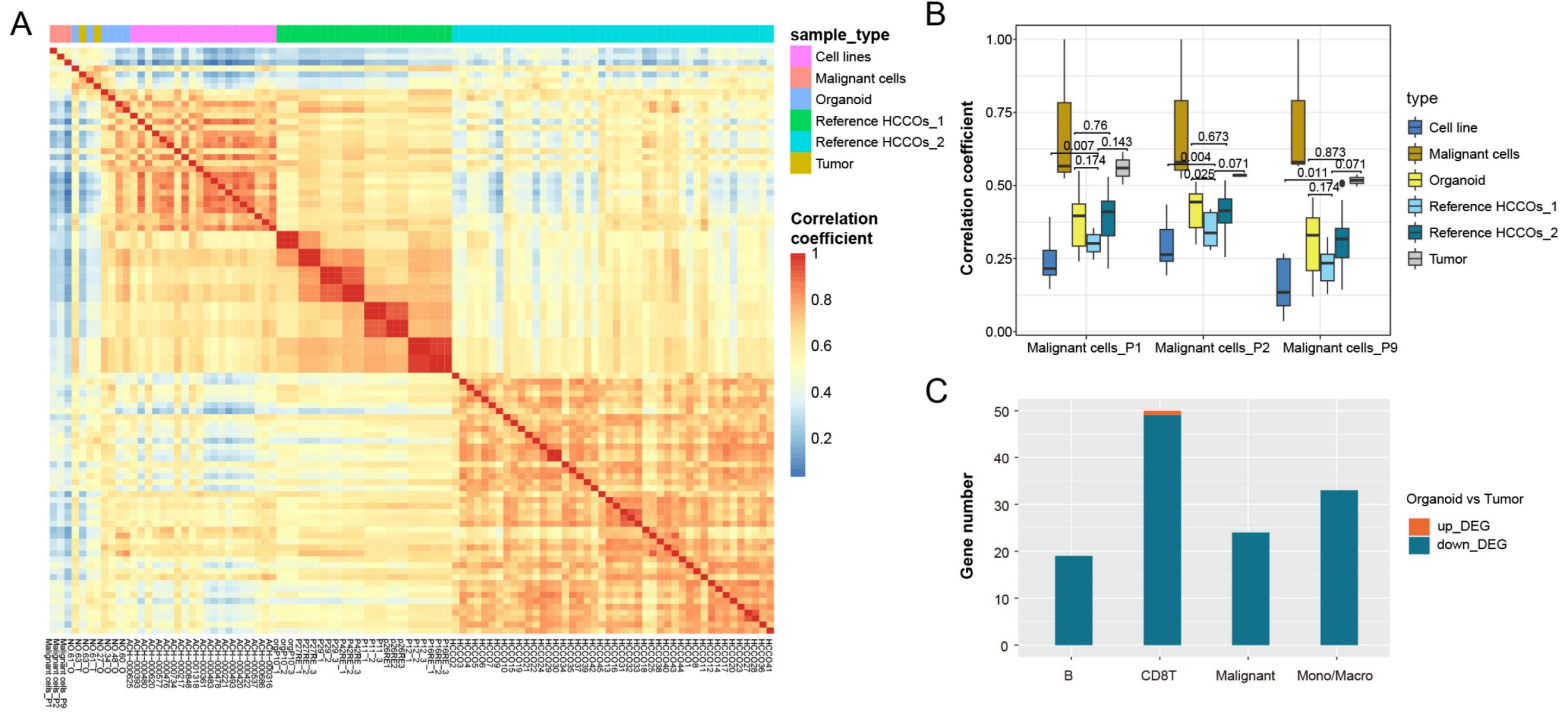
** Transcriptomic patterns of all expressed genes in PLCOs compared with cancer cell lines and tumor tissues.** (A) Heatmap of the expression pattern similarity of tumor-specific genes among samples. (B) Comparison of the similarity of the expression patterns of tumor-specific genes in various tissues compared to real tumor (malignant) cells. (C) The intersection between DEGs and cell type-specific genes.

Bulk sequencing obtains mean values of expression for all cells within a tissue, and direct comparison of expression patterns in tumor tissues and organoids is influenced by other cells in the tumor microenvironment. We therefore extracted tumor cell-specific genes from single-cell RNA-seq data to characterize these genes in tumor cells. We also analyzed other published HCCO data and cancer cell line data in combination with our HCCOs to compare our findings with real tumor cell expression patterns. Figure 4A shows the expression pattern similarity calculated based on tumor cell-specific genes from tumor cells in P1, P2, and P9. For the tumor cell-specific genes derived from real tumor cells, the expression patterns of tumor tissues were most similar to those of cancer cells and superior to those of HCCOs and cell lines (Figure 4B). Moreover, the similarity of our HCCOs with real tumor cells was significantly greater than that of HCCOs from reference cohort 1 and not inferior to that of HCCOs from reference cohort 2. Our HCCOs more closely resembled the expression pattern of real tumor cells than most published HCCOs, suggesting a more effective culture approach. Furthermore, the intersection of DEGs in PLCOs vs. tumor tissue and characteristic genes of cell types in the single-cell dataset was determined. The downregulated genes overlapped with genes characteristic of various immune cells, mainly CD8+ T cells and monocytes/macrophages, compared to those in tumor tissues (Figure 4C). Taken together, the immune cells in tumor tissues included mainly CD8+ T cells and monocytes/macrophages, which are the main sources of differences between tumor tissues and PLCOs.

### Identification of patient-specific drug sensitivity by PLCOs

**Figure 5:**
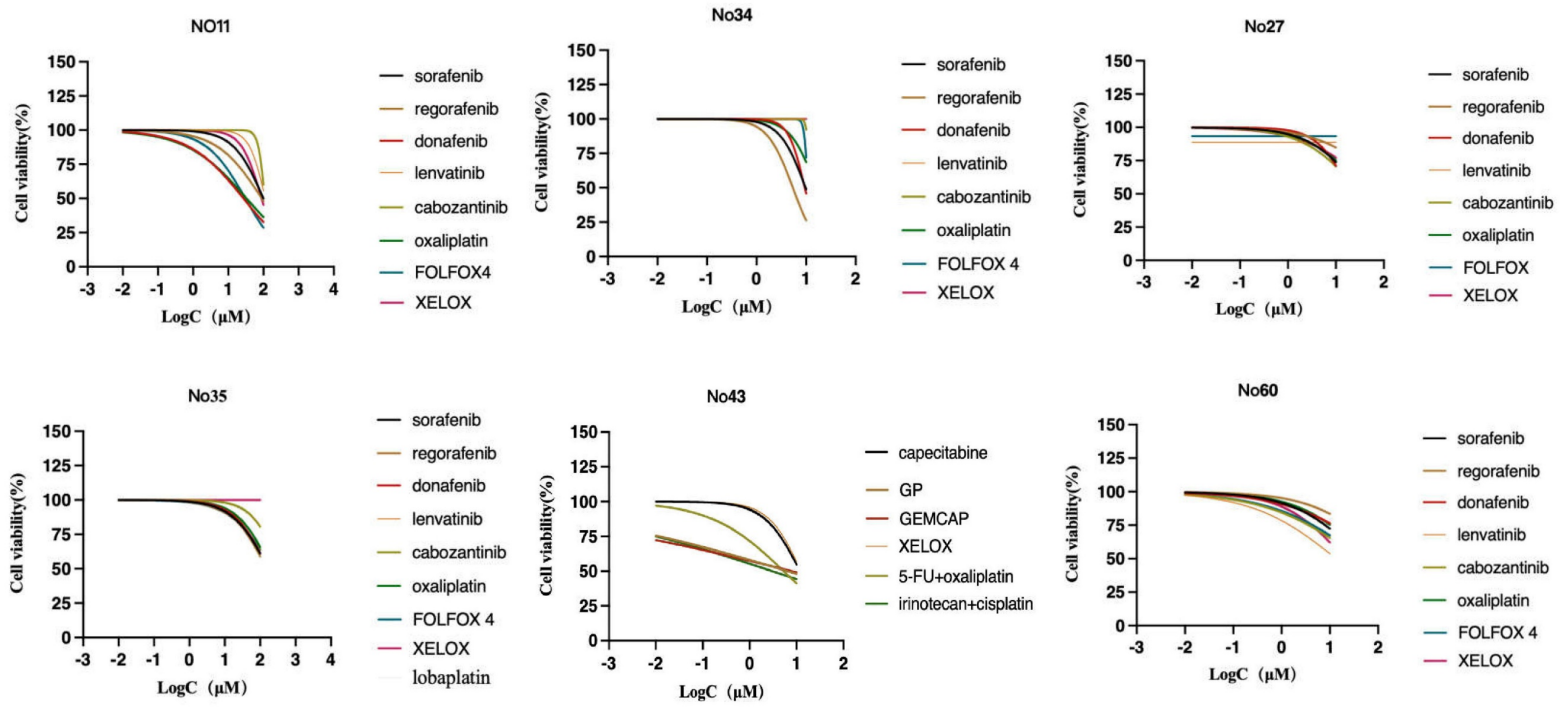
Dose-response curves after 6 days of treatment with PLC-related compounds generated from the luminescent signal intensities.

To assess patient-specific sensitivity, drug sensitivity testing was performed on 6 PLCOs to determine their utility. For each organoid, the sensitivity to multiple anticancer compounds in standard clinical care was evaluated. Before the measurement of cell viability, the PLCOs were processed for 6 days using a dilution series of each compound. The drug sensitivity for a subset of compounds was confirmed using an organoid formation assay based on the initial screen of prioritization, consequently verifying our screening technique. Based on the guidelines of the Chinese Society of Clinical Oncology (CSCO) for hepatocellular carcinoma (2022), systemic treatment was first and second-line recommended. Considering that PLCOs normally lack an immune microenvironment, we selected practical PLC-associated compounds or chemotherapy regimens in which differential sensitivity could be identified across PLCOs. For HCC, sorafenib, regorafenib, donafenib, lenvatinib, cabozantinib, oxaliplatin, FOLFOX4 (oxaliplatin, leucovorin, and 5-fluorouracil), XELOX (oxaliplatin and capecitabine), and lobaplatin (personalized medication of No. 35) were selected, whereas for ICC, capecitabine, GP (cisplatin and gemcitabine), GEMCAP (oxaliplatin and gemcitabine), XELOX, 5-fluorouracil plus oxaliplatin, and irinotecan plus cisplatin (personalized medication of No. 43) were selected. Drug sensitivity was represented as the half-maximal inhibitory concentration (IC50) (Table S3). We found that No. 35 was insensitive to all the tested compounds, whereas the other strains exhibited different responses to different drugs (Figure 5). Overall this drug screen fulfilled the requirements of selecting specific drugs for different patients with liver cancer, which was common in patient. Based on the molecular comparison results, the PLCO derived from patient No. 43 demonstrates a high level of concordance with the paired tumor tissue, both in terms of mutations and copy number alterations. Therefore, its drug testing results are more likely to simulate the patient's actual response.

**Figure 6:**
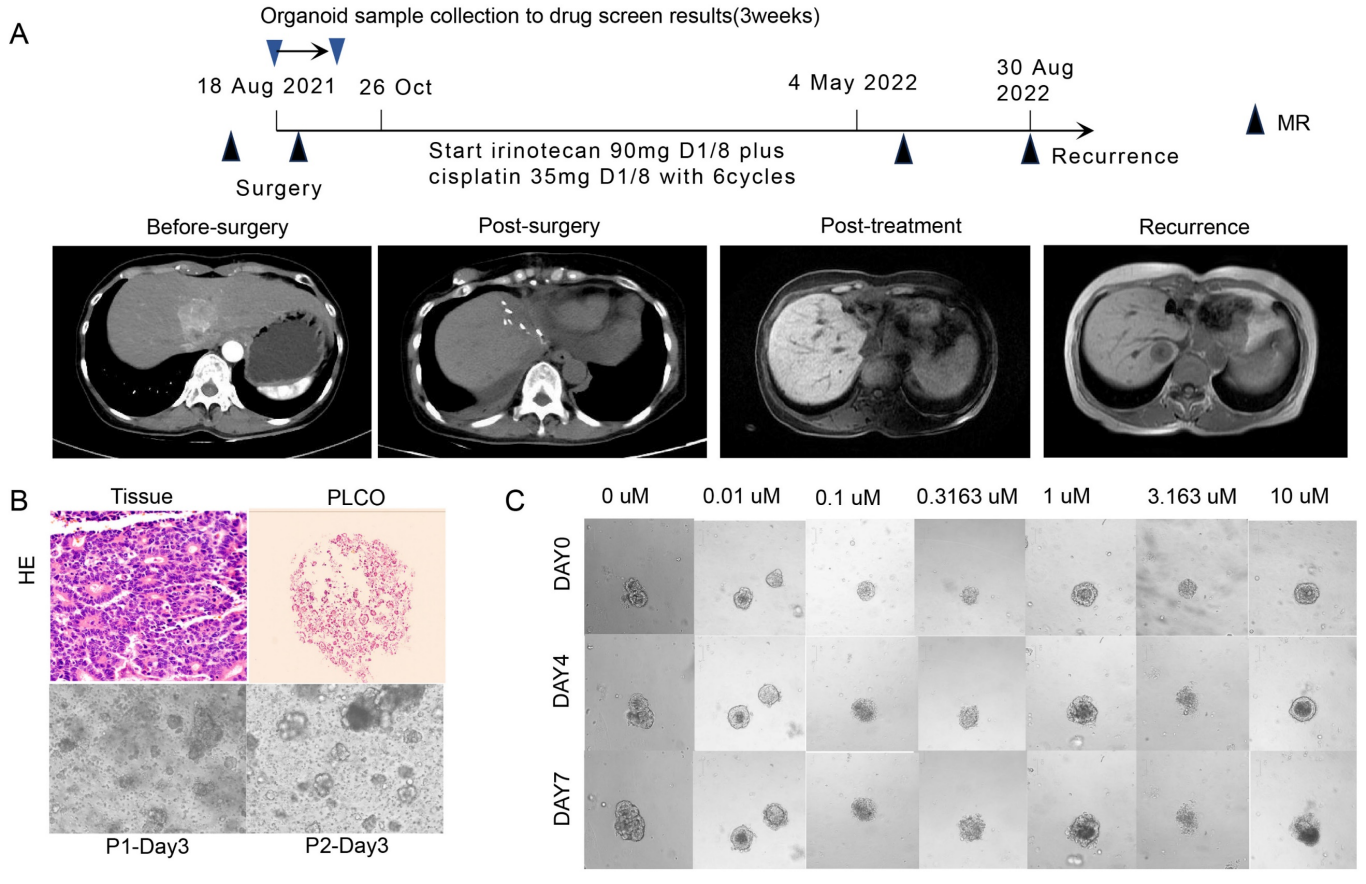
** PLCO reflects the clinical outcome of one patient with intrahepatic cholangiocarcinoma (ICC).** (A)Treatment and procedure timeline of the patient. The black triangle indicates the time point at which CT was administered, as indicated by the images in the middle row, and the red triangle indicates the time point at which PLCOs were obtained and the results of organoid drug sensitivity. (B) H&E staining of ICC tissue and PLCO (top row, scale bar:100 µm) and brightfield microscopy image of PLCOs in the first and second generations (P1 and P2) aon day 3 showing good growth of PLCOs. (C) Continuous brightfield microscopy image of PLCOs treated with different concentrations of irinotecan plus cisplatin at 0, 4, and 7 days.

A 60-year-old woman (No. 43) with a major complaint of relapse over 3 months after cholangiocarcinoma surgery visited our hospital. A partial hepatectomy was performed due to the presence of a space-occupying lesion alongside the second porta hepatis within the left hepatic lobe. Based on the postoperative histopathological results, cholangiocarcinoma accompanied by neuroendocrine carcinoma was diagnosed. Surgical tumor tissues were successfully obtained and cultured for PLCO, and the histochemical features of the tumor tissue were preserved (Figure 6A, B). In total, 6 chemotherapeutic regimens were evaluated as mentioned before, and PLCOs exhibited the greatest sensitivity to combined chemotherapy consisting of irinotecan plus cisplatin (IC50 of 3.03 µM) (Figure 6C; Figure S5). Therefore, adjuvant chemotherapy with irinotecan (90 mg, d1, d8) plus cisplatin (35 mg, d1, d8) was administered postoperatively. The patient developed diarrhea after first cycle of chemotherapy, leading to the discontinuation of irinotecan on day 8. After the first cycle, the patient's symptoms improved. The subsequent five cycles continued with irinotecan and cisplatin. After 2 cycles of chemotherapy, the patient experienced grade II myelosuppression, and recombinant human granulocyte colony-stimulating factors were subsequently administered. Finally, the CA-199 levels returned to normal after 6 cycles of chemotherapy. Postoperative CT revealed that the lesion did not expand. Altogether with the drug testing results and clinical practice guidelines, this patient was treated with irinotecan plus cisplatin postoperatively and showed a good clinical efficacy. The patient had recurrence one year after surgery (Figure 6A).

## Discussion

By establishing structures resembling adult organs, 3D cultures termed organoids have recapitulated the function and complexity of in-vitro mammalian tissues to some extent 9. In this study, we successfully established organoid cultures from 66 patients with PLC with the two most common PLC subtypes HCC and ICC and evaluated their consistency and the corresponding parental tumor tissues based on histopathology, genetic mutations, expression profiles, and drug sensitivity. Our results suggest that PLCOs can recapitulate the histological characteristics, genetic mutations, and expression profiles of original tumors well by preserving specific differences among patients, and among tumor subtypes. Moreover, PLCOs contributed to identifying patient-specific drug sensitivity. Importantly, few PLCO cultures have been established for drug testing, which allows the prediction of drug sensitivity in patient-specific patterns and the creation of personalized therapies. Because very few patients with liver cancer benefit from genetic testing, such as next-generation sequencing (NGS), PLCOs may serve as powerful preclinical tools for drug testing and personalized PLC treatment.

A distinct and key characteristic of PDO is that they can retain the genetic mutations of the original tumor tissue, which is highly distinct from existing 2D cell lines. In 3D culture, mammary epithelial cells are embedded in an extracellular matrix, allowing the cells to decipher the environment and self-composite into structures with tissue patterning, similar to that in the course of organogenesis. Furthermore, the cell extracellular interactions that occur in 3D culture can also restrain the anoikis apoptosis caused by detachment from the matrix of cancer cells without causing any changes to survive in an environment with no extracellular matrix, consequently promoting the preservation of heterogeneous populations in culture 27. The PLCOs established in this study further verified the similarities and differences between the organoids and the original tumors. They could recapitulate the histological architecture and genetic and transcriptomic traits of the parental tumor tissue and maintain the specific differences between patients and tumor subtypes.

Because of the preservation of intratumor heterogeneity and the tumor microenvironment, organoids can be more appropriate for drug screening than can cell lines 28, 29. In addition, they are more efficient and less expensive than murine tumor models 3. In culture, capturing genetic changes in original tumors has made the use of PDO a potentially preclinical approach for screening drug sensitivity and identifying novel therapeutic targets, thus facilitating the personalized management of liver cancer. Broutier et al. reported that PDOs from liver tumors are sensitive to ERK inhibition which was verified in xenograft models 11. Chen et al. reported that the immunosuppressant mycophenolic acid effectively suppressed the initiation and growth of mouse liver organoids 30. In this study, we found good consistency between the screening and validation results, and a patient achieved a durable response after the use of combined chemotherapy consisting of irinotecan plus cisplatin based on the tdrug sensitivity results.

One of the major strengths of this study was that the expression patterns of PLCOs were first analyzed at the single-cell level, which revealed the superiority of organoids over cell lines in maintaining cancer cell characteristics. Importantly, a typical case reported in our study clinically demonstrated the ability of organoids to identify patient-specific drug sensitivity. With an improved 2:2 method and a large cohort, the success rate for organoid generation was 40.9%, which is relatively high compared with that of previous studies. However, this rate is still lower than the previously reported success rates for colorectal cancer (90%) and pancreatic cancer (75%-83%) 31, 32. This lower success rate may be partially because the characteristics of epithelial stem cells required for propagation in the organoid culture system were absent in hepatocytes, which are the cells of origin 33. Furthermore, in addition to chemotherapy and multikinase inhibitors, such as sorafenib, the Food and Drug Administration has approved nivolumab, pembrolizumab, ramucirumab, nivolumab/ipilimumab, atezolizumab/bevacizumab, and tremelimumab/durvalumab, as first- or second-line monoclonal antibodies (mAbs) for unresectable HCC. However, information on the immune and vascular microenvironments is lacking in organoid culture systems, which is crucial for testing these drug responses in tumors 11, 34. Therefore, the use of organ-on-chips with an immune microenvironment or vascularization can be beneficial for further testing more drugs for liver cancer treatment in the future. The combination of organoids and comprehensive genomic profiling, from genomic information to phenotypic drug sensitivity results, can help to comprehensively select the appropriate treatment plan for patients, which can be the real trend of future individualized treatment.

## Conclusions

The PLC-derived organoids developed in this study can highly recapitulate the characteristics of the two most common subtypes of liver cancer, including histological featuresand genetic and transcriptomic traits, and can help determine patient-specific drug sensitivity. Therefore, PLC-derived organoids can be considered a potential therapeutic approach for drug testing and personalized PLC treatment.

## Supplementary Material

Supplementary figures and tables.
