# Supplementary material for: Leveraging Patient-Derived Organoids for Personalized Liver Cancer Treatment
Source: Int J Biol Sci. 2024 Oct 7;20(13):5363–74. doi: 10.7150/ijbs.96317 (PMC11488587; doi:10.7150/ijbs.96317)
Supplement: Supplementary file 1 — Supplementary figures and tables. [file ijbsv20p5363s1.pdf]

Supporting Information

Leveraging Patient-Derived Organoids for Personalized Liver Cancer Treatment

Jianhua Rao[1,#], Chao Song[2,4,#], Yangyang Hao[3,#], Zaozao Chen[2,6,8,#], Sidu Feng[3], Shihui Xu[6], Xiaoyue Wu[7], Zhengfeng Xuan[1], Ye Fan[1], Wenzhu Li[1], Junda Li[1], Yong Ren[5,*], Jian Li[3,*], Feng Cheng[1,*], Zhongze Gu[2,4,6,7,8,*]

[1]Hepatobiliary Center of The First Affiliated Hospital, Nanjing Medical University; Research Unit of Liver Transplantation and Transplant Immunology, Chinese Academy of Medical Sciences, Nanjing, China

[2]State Key Laboratory of Digital Medical Engineering, Southeast University, Nanjing, China

[3]Key Laboratory of DGHD, MOE, School of Life Science and Technology, Southeast University, Nanjing, China

[4]School of Biological Science & Medical Engineering, Southeast University, Nanjing, China

[5]State Key Laboratory of Translational Medicine and Innovative Drug Development, Jiangsu Simcere Diagnostics Co, Nanjing, China

[6]Jiangsu Avatarget Co, Suzhou, China

[7]Jiangsu Institute for Health and Sport (JIHS), Nanjing, China

[8]Institute of Medical Devices (Suzhou), Southeast University, Nanjing, China

[#]These authors are co-first author and equally contribute to this work.

[*]Correspondence: Yong Ren, State Key Laboratory of Translational Medicine and Innovative Drug Development, Jiangsu Simcere Diagnostics Co, Nanjing, China. Email: yong.ren@simceredx.com;

Jian Li, Key Laboratory of DGHD, MOE, School of Life Science and Technology, Southeast University, Nanjing, China. Email: jianli2014@seu.edu.cn;

Feng Cheng, Hepatobiliary Center of The First Affiliated Hospital, Nanjing Medical University; Research Unit of Liver Transplantation and Transplant Immunology, Chinese Academy of Medical Sciences, Nanjing, China. Email: docchengfeng@njmu.edu.cn;

Zhongze Gu, State Key Laboratory of Digital Medical Engineering, Southeast

University, Nanjing, China. Email: gu@seu.edu.cn

Supplementary Figures

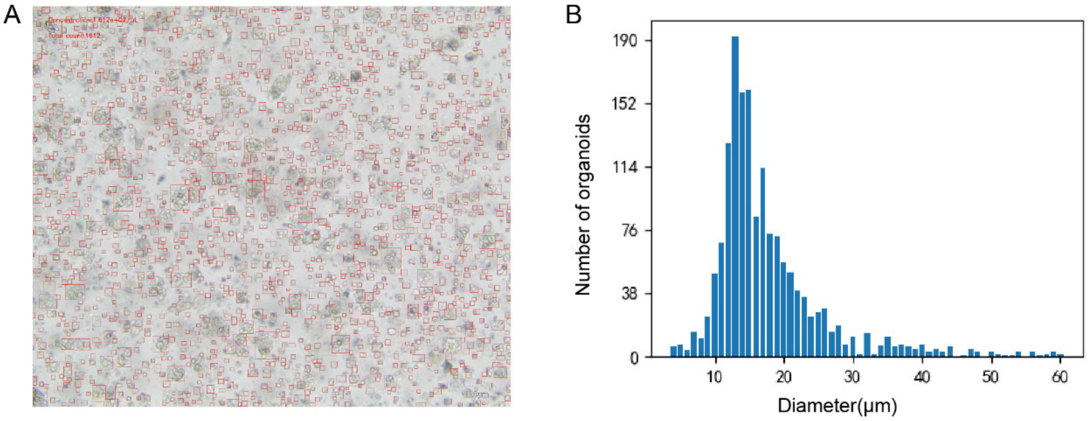

Figure S1. Seeding after digestion during organoid passage. (A) Visualization of organoid recognition results. (B) Organoid size distribution. This well demonstrates the distribution of organoids based on their diameters. It shows that the organoids range in size from 4 to 60 micrometers, with a mode around 13 micrometers and a median near 16 micrometers.

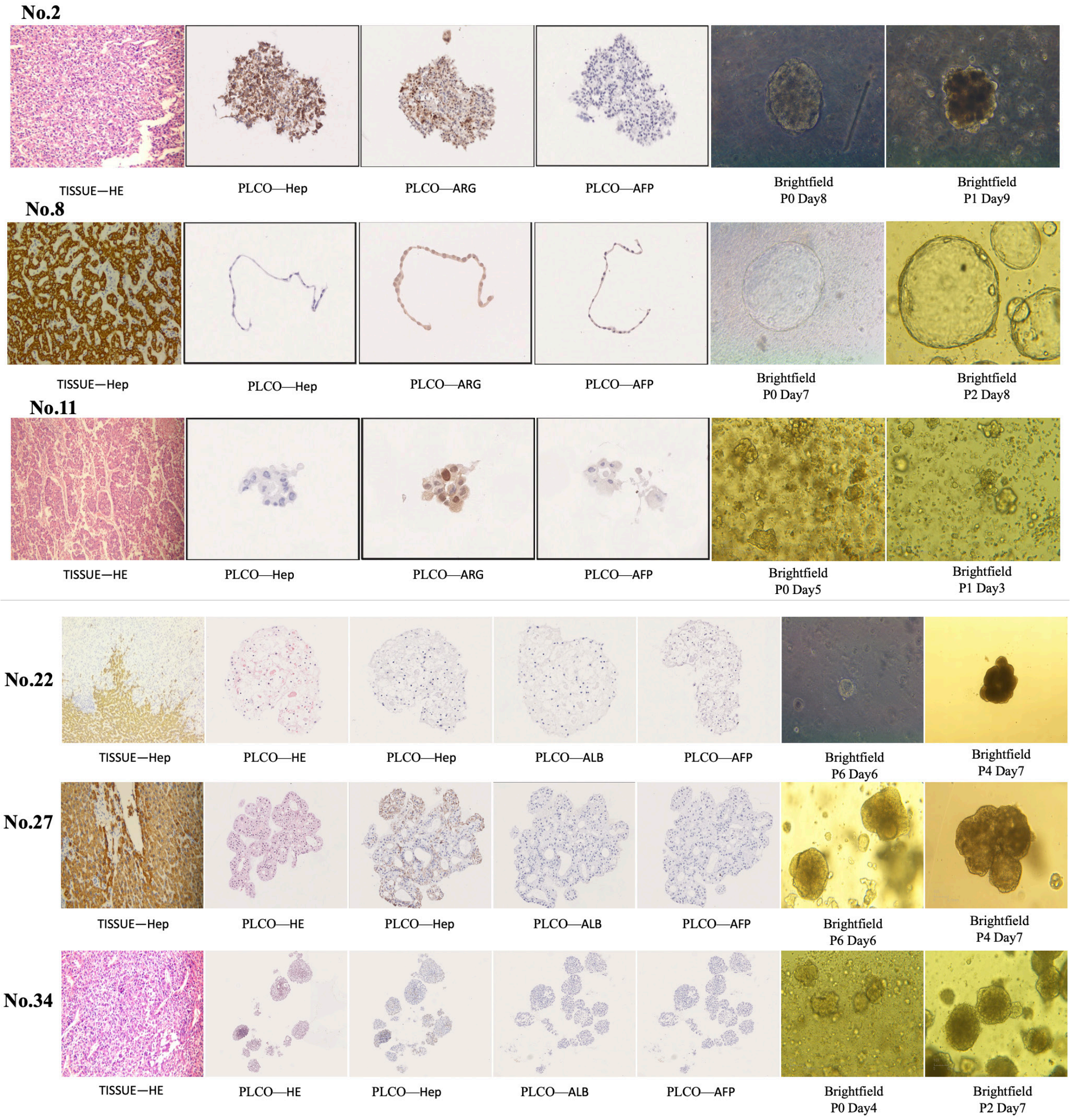

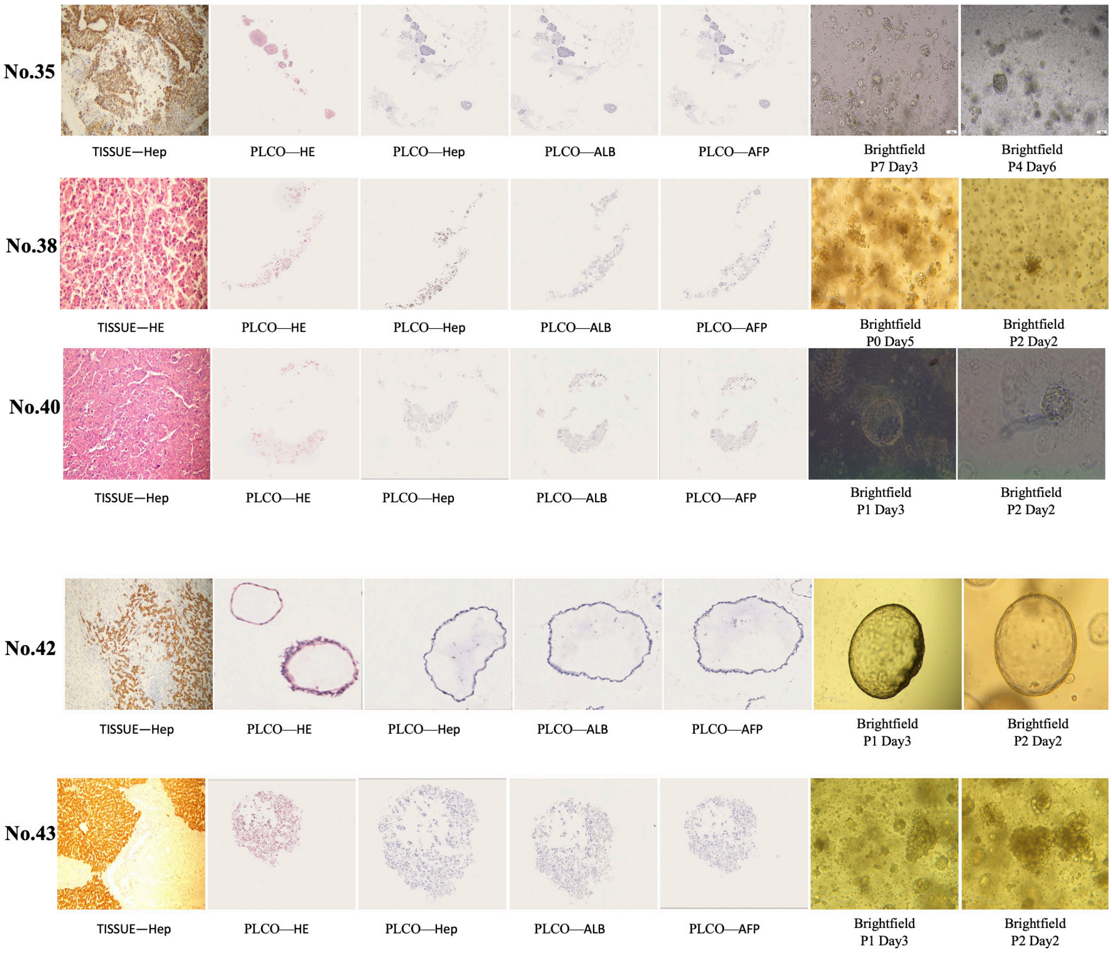

Figure S2. Bright-field images of all successfully cultured organoids, along with corresponding IHC staining for Hep, ARG, ALB, and AFP and Tissue HE or Hep.

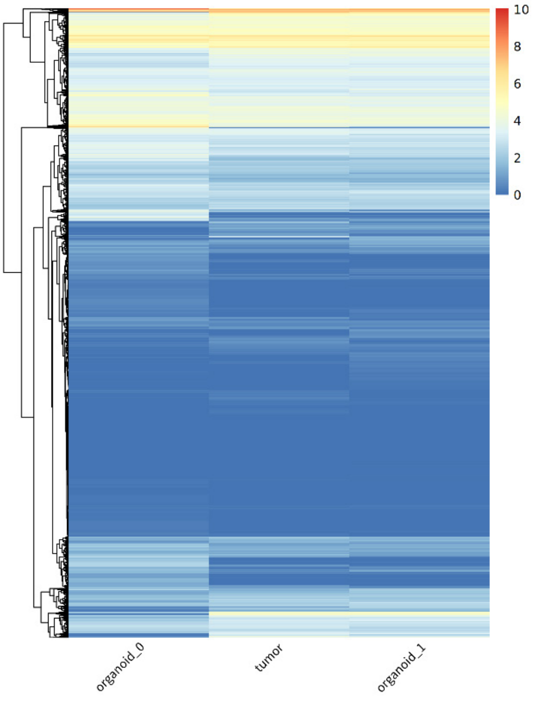

Figure S3. Cluster analysis was used to compare the gene expression patterns of organoids and tumor tissues cultured under different experimental conditions, in which genes were clustered according to the similarity of expression. organoid_0, organoid_1, and tumor, denote the organoids cultured before medium optimization, the organoids cultured after medium optimization, and the corresponding tumor tissues, respectively.

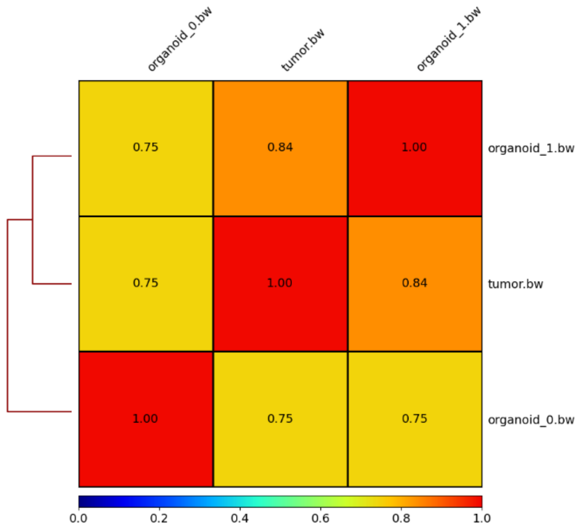

Figure S4. Quantitative assessment of gene expression similarity between organoid_0, organoid_1, and tumor. The closer the correlation coefficient is to 1, the higher the similarity of expression patterns between the samples.

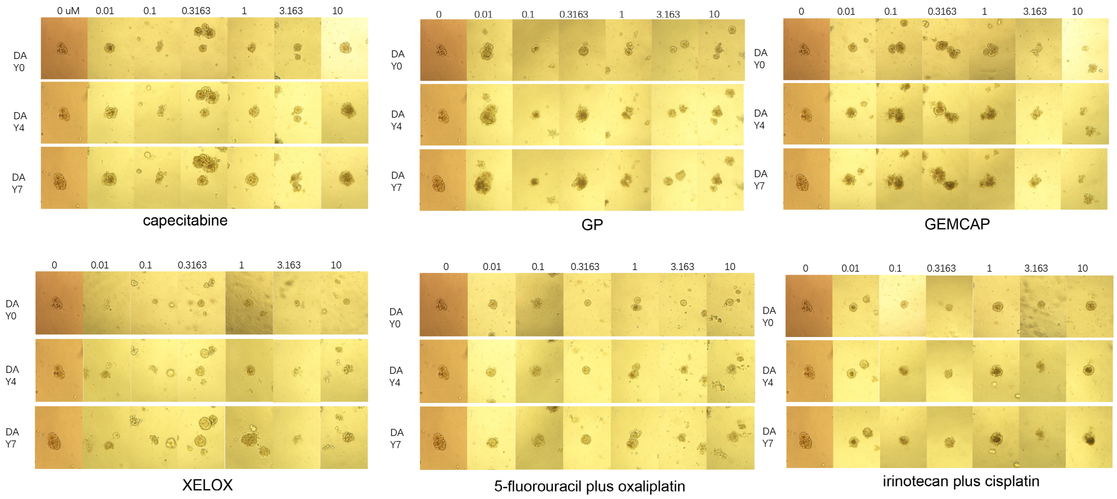

Figure S5. Effect of different compound concentrations on organoid formation at different time points in the No. 43 sample.

Supplementary Tables

Table S1 Key resources table.

REAGENT or RESOURCE	SOURCE	IDENTIFIER
Liver tumor organoid culture kit	Jiangsu Avatarget Biotechnology	Cat#KLV0101
Matrigel	Corning	Cat#356231
Liver tumor organoid medium	Jiangsu Avatarget Biotechnology	Cat#MLV0101
TrypLE Express	ThermoFisher	Cat#12605010
Phosphate buffer saline (PBS)	Solarbio	Cat#P1020
Nicotinamide	Sigma-Aldrich	Cat#98-92-0
Rspo-1	MCE	Cat#HY-P7114
BMP4	MCE	Cat#HY-P7007

Table S2 The baseline characteristics of 19 patients.

Number	Gender	Age	Pathological types	HBV/HCV infection	TNM staging
No.8	Male	63	ICC	No	
No.11	Male	58	HCC	No	
No.18	Female	58	Other	No	
No.27	Female	65	HCC	Yes	II
No.34	Male	54	HCC	Yes	III
No.35-1/2	Male	57	HCC	No	III
No.37	Female	64	HCC	No	III
No.38	Male	62	HCC	No	II
No.40	Male	51	HCC	No	II
No.42	Male	67	ICC	No	
No.43	Female	59	ICC	No	II
No.48	Male	64	HCC	No	
No.49	Female	65	Other	Unknown	III
No.54	Male	58	HCC	No	
No.55	Female	48	HCC	No	IV
No.57	Male	73	Other	No	
No.60	Female	70	HCC	No	
No.61	Male	56	HCC	No	III
No.63	Female	68	HCC	No	IV

Abbreviations: ICC, intrahepatic cholangiocarcinoma; HCC, hepatocellular carcinoma; HBV/HCV, hepatitis B/C virus.

Table S3 The IC50 values for all drugs in six PLCOs..

Drugs	No.11	No.27	No.34	No.35	No.43	No.60
sorafenib	99.86	>10	9.745	>100	ND	>10
regorafenib	89.85	>10	5.390	>100	ND	>10
donafenib	26.98	>10	9.391	>100	ND	>10
lenvatinib	>100	>10	>10	>100	ND	>10
cabozantinib	>100	>10	>10	>100	ND	>10
oxaliplatin	33.05	>10	>10	>100	ND	>10
FOLFOX 4	30.63	>10	>10	>100	ND	>10
XELOX	88.43	>10	>10	>100	>10	>10
lobaplatin	ND	ND	ND	>100	ND	ND
capecitabine	ND	ND	ND	ND	>10	ND
GP	ND	ND	ND	ND	6.426	ND
GEMCAP	ND	ND	ND	ND	7.966	ND
5-fluorouracil + oxaliplatin	ND	ND	ND	ND	5.298	ND
irinotecan + cisplatin	ND	ND	ND	ND	3.029	ND

μM	1-10	> 10	10-50	50-100	> 100	NOT DONE
